# Expression, secretion and surface display of a human alkaline phosphatase by the ciliate *Tetrahymena thermophila*

**DOI:** 10.1186/1472-6750-11-11

**Published:** 2011-01-31

**Authors:** Ingo Aldag, Ulrike Bockau, Jan Rossdorf, Sven Laarmann, Willem Raaben, Lutz Herrmann, Thomas Weide, Marcus WW Hartmann

**Affiliations:** 1 Cilian AG, Johann-Krane-Weg 42, D-48149 Münster, Germany; 2 University Hospital Münster, Department of Internal Medicine D, Division of Molecular Nephrology, Domagkstr. 3a, 48149 Münster, Germany; 3 Carl Zeiss MicroImaging GmbH, Königsallee 9-21 37081 Göttingen, Germany; 4 AM-Pharma Holding BV, Rumpsterweg 6, 3981 AK Bunnik, The Netherlands; 5 Evonik Degussa, GmbH Paul-Baumann-Str. 14, 5772 Marl, Germany

## Abstract

**Background:**

*Tetrahymena thermophila *possesses many attributes that render it an attractive host for the expression of recombinant proteins. Surface proteins from the parasites *Ichthyophthirius multifiliis *and *Plasmodium falciparum *and avian influenza virus antigen H5N1 were displayed on the cell membrane of this ciliate. Furthermore, it has been demonstrated that *T. thermophila *is also able to produce a functional human DNase I. The present study investigates the heterologous expression of the functional human intestinal alkaline phosphatase (hiAP) using *T. thermophila *and thereby presents a powerful tool for the optimization of the ciliate-based expression system.

**Results:**

Functional and full length human intestinal alkaline phosphatase was expressed by *T. thermophila *using a codon-adapted gene containing the native signal-peptide and GPI (Glycosylphosphatidylinositol) anchor attachment signal. HiAP activity in the cell extract of transformants suggested that the hiAP gene was successfully expressed. Furthermore, it was demonstrated that the enzyme was modified with N-glycosylation and localized on the surface membrane by the C-terminal GPI anchor. A C-terminally truncated version of hiAP lacking the GPI anchor signal peptide was secreted into the medium as an active enzyme. In a first approach to establish a high level expression system up to 14,000 U/liter were produced in a time frame of two days, which exceeds the production rate of other published expression systems for this enzyme.

**Conclusions:**

With the expression of hiAP, not only a protein of commercial interest could be produced, but also a reporter enzyme that offers the possibility to analyze *T. thermophila *genes that play a role in the regulation of protein secretion. Additionally, the fact that ciliates do not secrete an endogenous alkaline phosphatase provides the possibility to use the truncated hiAP as a reporter enzyme, allowing the quantification of measures that will be necessary for further optimization of the host strains and the fermentation processes.

## Background

The ciliate *Tetrahymena thermophila *is a small non pathogenic protozoan and one of the best characterized eukaryotic unicellular organisms. It has been used as a model system for basic molecular-, cell- and developmental biology research for decades [1-6].

Furthermore, it has been shown that *T. thermophila *is able to grow fast to high cell densities in cheap medium in a common bioreactor infrastructure [7,8]. The beneficial growth characteristics combined together with the sub-cellular machinery for performing eukaryotic post-translational protein modifications and the established genetic engineering technology makes *T. thermophila *a potential alternative to more established expression systems [6-9]. It has been reported that this system is capable of expressing GPI linked surface proteins from the parasitic ciliate *Ichthyophthirius multifiliis*, the malaria parasite *Plasmodium falciparum *and avian influenza virus antigen H5N1, suggesting that *T. thermophila *could play an important role in vaccine development [10-13]. Moreover, we could show that *T. thermophila *is also able to express and secrete the functional human enzyme DNase I [14].

The major challenge now is the transition of the *Tetrahymena *based expression system from laboratory model to useful production platform. It is therefore important to generate improved *T. thermophila *strains for maximizing the production yield and for meeting the specific requirements of a recombinant protein for a particular application. This can be achieved by studying the processes that regulate synthesis, post-translational modification and sorting of recombinant proteins and by understanding how they can be controlled. A further objective is the establishment of optimized fermentation procedures. To date, upstream efforts have focused primarily on the improvement of growth conditions [7-9,15]. Little, if any, attention has been paid to determine cultivation parameters that enhance the productivity of recombinant proteins.

The availability of a robust reporter enzyme that allows to evaluate the efficiency of protein targeting on the one hand and to define optimal upstream processes on the other hand would be very benefical to attain this goal.

Here we report the expression and secretion of the human intestinal alkaline phosphatase that can be used as a tool for the development of a high-yield glycoprotein expression system. So far several alkaline phosphatases (EC 3.1.3.1) of mammalian cells have been characterized. These enzymes are divided into tissue-specific (derived from intestine, placenta or germ cells) and tissue non-specific (derived from liver, bone and kidney) alkaline phosphatases [16].

The hiAP precursor protein consists of 528 amino acids (aa), including a signal peptide at its N-terminus (aa 1-19) and a GPI anchor signal (aa 504-528) at its C-terminus. The mature and processed enzyme lacking the first 19 and last 25 amino acids is localized on the cell surface due to a post-translational added GPI anchor. It is postulated that the mature protein contains three potential N-glycosylation sites and two disulfide bridges (aa 140 to 202; aa 486 to 493) [17,18].

We prepared two constructs encoding the precursor hiAP with and without a GPI anchor signal. In order to avoid problems in expression due to the unique codon bias of ciliates a synthetic, codon-adapted gene, in which all of the very rare codons were replaced by highly frequented codons, was used. We demonstrated that both variants could be expressed as functional and glycosylated proteins. Furthermore, we used the C-terminal truncated form of human intestinal alkaline phosphatase that lacks the GPI anchor signal in first optimization experiments.

## Results

### Expression and localization of human intestinal alkaline phosphatase in *T. thermophila*

**Figure 1:**
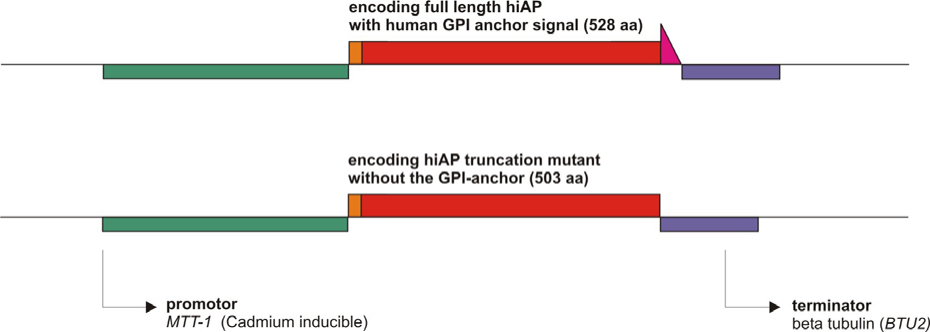
**Structure of the used expression cassettes**. Two expression cassettes were used in this study. The first expression cassette encodes the full-length hiAP precursor protein (aa 1- 528), including the N-terminal ER leader sequence (aa 1-19) and the C-terminal GPI anchor/cleavage site (aa 504-528). The second cassette encodes hiAP (aa 1- 503) without the GPI anchor/cleavage signal, consequently no GPI moiety can be added to the recombinant enzyme. The synthetic hiAP cDNA was flanked by a ~1 kb *MTT1 *promoter active sequence and the *BTU2 *terminator (~350 bp).

In order to express the full-length precursor of hiAP in ciliates we used a codon adapted artificial gene, according to the codon bias for highly expressed genes in *T. thermophila *[19,20]. The complete hiAP cDNA was cloned into the rDNA ori based pH4T2 vector [21,22]. The expression of the hiAP was regulated by the *MTT1*-promoter of *T. thermophila *[23]. This promoter system can be induced by the addition of cadmium ions to the culture medium (see Figure 1).

**Figure 2:**
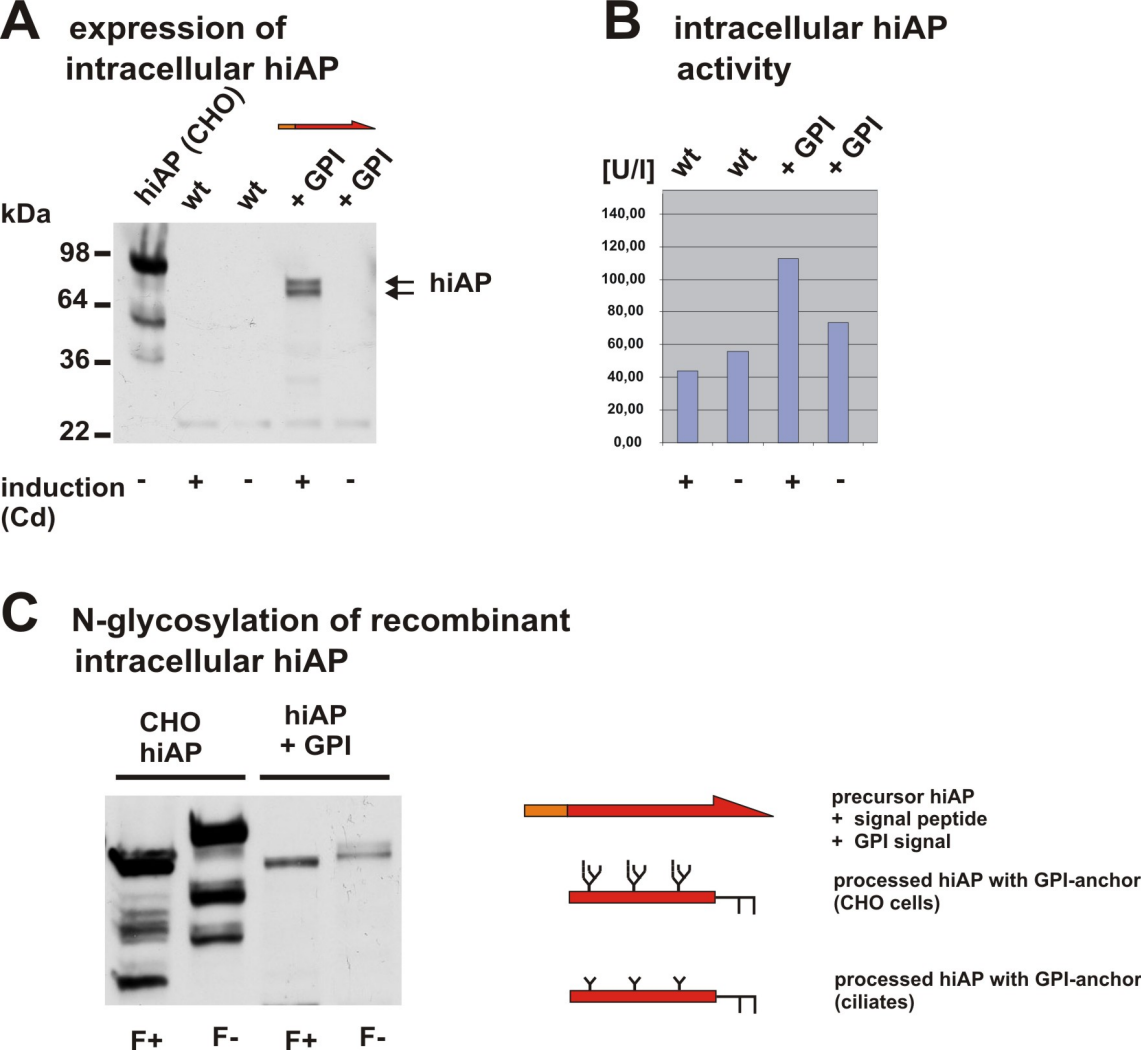
**Characterization of recombinant full-length hiAP**. A: Western blot analysis of cell extracts. In a positive clone hiAP expression was induced by the addition of cadmium to the medium. Extracts from induced cells showed a hiAP signal in the Western blot. Extracts from wild type cells (induced and non-induced) and the non-induced cells of the positive clone showed no signal. An extract from transformed CHO cells served as positive control. The double band probably corresponds to intracellular precursor hiAP with N-terminal signal peptide or to a non-cleaved GPI anchor signal. **B**: The data shown in the Western blot were confirmed by an alkaline phosphatase activity assay. The samples derived from wild type cells treated with and without cadmium (wt +Cd; wt -Cd) and extracts from the non-induced hiAP clone (+GPI -Cd) showed only basal activity. In contrast to this an elevated enzyme activity could be observed in cell extracts from hiAP expressing cells (+GPI +Cd), suggesting that recombinant hiAP is expressed as an active enzyme. **C**: We treated extracts of the hiAP expressing cells with the enzyme PNgase F (F+). Extracts from CHO cells expressing hiAP were used as a positive control. Negative controls were non-treated cell extracts (F-). The results show a significant band shift, indicating that both CHO derived as well as *T. thermophila *derived hiAP carries N-glycans. As expected, the band shift in ciliates is less significant due to the smaller N-glycan structure. In contrast to mammalian proteins that carry a complex N-glycosylation *T. thermophila *has most probably an N-glycan structure of the oligo-mannose Man_3_GlcNAc_2 _type (see scheme).

We transformed conjugating *T. thermophila *cells. Clones that were resistant against paromomycin were analyzed further. Cadmium was added to putative positive clones to induce hiAP expression and the cell extracts were analyzed by Western blot using a specific antiserum from sheep (L-19, Santa Cruz Biotechnology) originally derived against an internal epitope of human placental and intestinal alkaline phosphatase. The non-induced cells of these clones and a wild type *T. thermophila *strain served as negative controls. Cell extracts of transformed CHO cells containing the full-length hiAP were used as positive control. Figure 2 shows that the antibody specifically recognizes one single protein with the apparental molecular weight of ~60 kDa, indicating that recombinant hiAP was expressed by the transformed and cadmium treated ciliates.

HiAP has three putative N-glycosylation sites at position, N_141_, N_268 _and N_429_. To analyze whether or not recombinant hiAP from *T. thermophila *is glycosylated during its passage through the ER and Golgi membranes we performed a de-glycosylation assay, by using PNGase F (N-Glycosidase F). This enzyme is an amidase that cleaves between the amino acid asparagine (N) and the innermost N-acetylglucosamine residue (GlcNAc) of high-mannose, hybrid and complex oligo-saccharides from N-linked glycoproteins. We observed a significant reduction of the molecular weight of recombinant hiAP from CHO cells (control) as well as of hiAP derived from ciliates in samples that were treated with PNGase F (see Figure 2C). These results indicate that N-glycans were present in both samples. The elimination of the N-glycan structure of hiAP derived from *T. thermophila *leads to a smaller band shift than the de-glycosylation of the protein expressed by CHO cells. This might result from a distinct bias of site occupancy, but previous data showed that the band shift difference was referable to a differential N-glycosylation pattern. N-glycan moieties in *T. thermophila *were almost completely of the oligo-mannose type [14], while the oligosaccharide structure of proteins from CHO cells is of the hybrid or complex structure. The de-glycosylated form of recombinant hiAP derived from CHO and ciliates had the same molecular weight, suggesting that the recombinant hiAP enzyme is properly expressed. To confirm the expression of a functional enzyme we prepared crude cell extracts of induced, non-induced and wild type cells and tested them for alkaline phosphatase activity. The enzyme activity in extracts of cadmium induced cells was compared to the activity of extracts from non-induced and wild type cells. We observed elevated phosphatase activity in the cells that were also positive in the Western blot analysis, suggesting that hiAP becomes expressed as an active, correctly folded enzyme.

**Figure 3:**
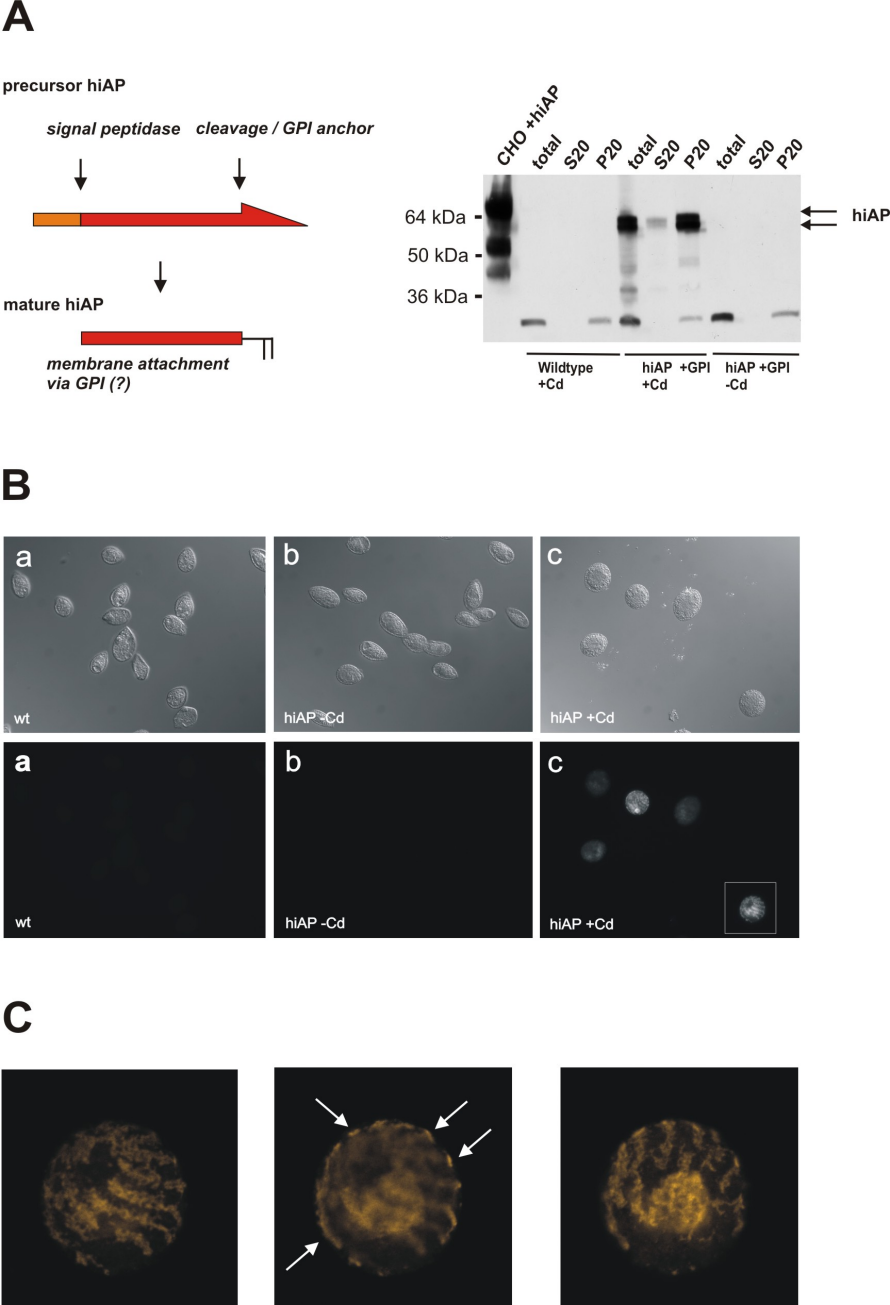
**Localization of recombinant hiAP in *T. thermophila *cells**. **A**: Cell fractionation experiment: Cell extracts were prepared from wild type cells and hiAP expressing cells treated with cadmium (+Cd) and without cadmium (-Cd). The whole protein preparation (total) was done without detergent and subsequently centrifuged at 20,000 × g. The insoluble fraction (pellet, P20) and the soluble fraction (supernatant, S20) were analyzed by SDS-PAGE and compared to the total fraction. Almost all of the recombinant hiAP (arrows) was found in the insoluble cell fraction, suggesting that most of the recombinant protein is proper processed and therefore membrane associated. The double band probably corresponds to intracellular precursor hiAP that is membrane attached by the signal peptide or corresponds to a non-cleaved GPI anchor signal. The scheme on the left side illustrates the processing of precursor hiAP into the mature enzyme. **B**: Immunofluorescence analysis: In order to confirm the cell fractionation data we performed a microscopic analysis. *T. thermophila *cells that express hiAP and negative controls (wild type and non-induced hiAP cells) were fixed and subsequently stained by the L-19 antiserum. The upper panel shows the Nomarski images (control) of the stained cells in the lower panel. The figure clearly illustrates that only induced cells lead to a significant staining. The cell marked with a white box was analyzed in a higher magnification. **C**: Detailed study of image B-c: HiAP expressing cells have a distinct staining pattern. A confocal scan through the cell demonstrated that the main hiAP signals are found on the surface of the ciliate cell (arrows). Additional signals were found in the middle of the cells. This not further characterized structure probably corresponds to transport vesicles or membranous structures that carry precursor forms of the recombinant protein. Further complete detail scans of hiAP expressing cells are shown in the two Additional files 1 and 2.

In human tissues alkaline phosphatases are membrane associated by C-terminal GPI anchor moieties and localized on the cell surface [16-18]. To monitor the functionality of the human GPI anchor signal in the ciliate *T. thermophila*, we analyzed the localization of recombinant hiAP in ciliates by two independent approaches. First, we made a simple fractionation experiment. Extracts from cadmium-induced cells were lyzed using a buffer without detergent (total) and centrifuged at 20,000 × g. The basic cell extract (total), the supernatant (S20) and the pellet fraction (P20) were analyzed by SDS-PAGE and Western blot analysis. Figure 3A illustrates that almost all of the recombinant hiAP was found in the P20 fraction, suggesting that the recombinant hiAP is membrane associated and not a soluble protein.

Secondly, by using confocal microscopic analysis, we were able to confirm these data. The top panel of Figure 3B shows the Normasky images and the bottom the immuno staining of *Tetrahymena *cells. The images show that the antibody L-19 specifically recognized a protein in only those clones in which the expression was induced by the addition of cadmium (Figure 3B-c, bottom). The wild type cells (Figure 3B-a) and the non-induced cells (Figure 3B-b) did not show signals above background. Surprisingly, the staining shows a distinct pattern and no continuous staining. This pattern is shown in Figure 3C at higher magnification. A more detailed scanning analysis using 0.50 μm scanning steps is shown in Additional file 1, Figure 1 and Additional file 2, Figure 2. These figures clearly illustrate that recombinant hiAP predominantly localizes to the surface of the cells. Additional structures stained within the cell probably correspond to transport vesicles and/or to intracellular membranous structures that contain hiAP precursor proteins. The previously shown localization of the heterologous expressed surface protein CSP-1 a GPI anchored surface protein of *Plasmodium falciparum *indirectly confirms this thesis because the immunofluorescence pattern of this protein shows significant similarities to hiAP localization [11]. These results suggest that the human full-length hiAP protein is correctly processed and targeted in the ciliate system.

### Secretion of functional hiAP into the medium

**Figure 4:**
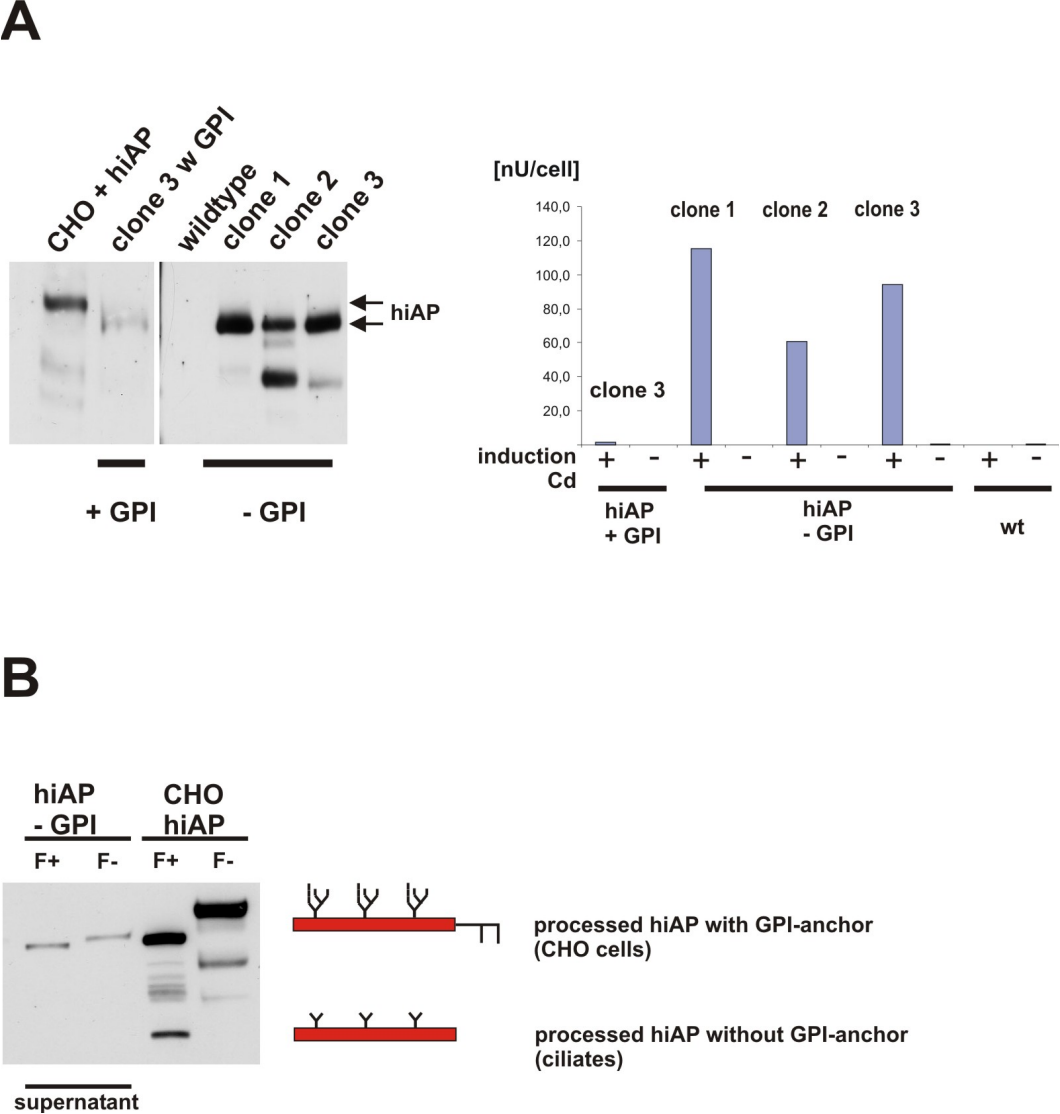
**Secretion of hiAP that lacks the GPI anchor signal**. **A**: Expression and secretion of functional recombinant hiAP. We analyzed the supernatant of three clones that were induced with cadmium. The clones carried the expression cassette that encodes hiAP without the GPI anchor signal. As control we used the clone shown in Figure 2 (clone 3 +GPI), the wild type and an extract from CHO cells that express hiAP. Clone 1, 2 and 3 show a positive signal in the Western blot analysis, the additional bands are due to protein degradation (clone 2 and 3). As expected almost no hiAP expression signal was observed in the supernatant of cells expressing hiAP +GPI (clone 3 +GPI). The enzyme activity assay (right side of the figure) confirms the data of the Western blot analysis. Clone 2 and 3 are positive, but the highest activity was found in the supernatant of clone 1. No or only few enzyme activity was found in supernatants of the wild type and in the supernatant of the clone that expresses full-length hiAP. The activity is given in nU/cell (measured activity per cell) to allow a direct comparison between the supernatant of the different clones. **B**: N-glycosylation of secreted hiAP. We treated the supernatant of one clone with PNGase F and analyzed the samples by SDS-PAGE and Western blot. As a control we used the extract from CHO cells that express hiAP. We found a significant band shift, suggesting that also truncated hiAP becomes glycosylated during the passage through the ER and Golgi compartments like the full-length hiAP protein. The band shift in the ciliate samples is less significant due to the smaller oligo-mannose N-glycan structure. The scheme illustrates the different post-translational modification in ciliates compared to the CHO cells.

In the next experiments we used an expression cassette encoding a hiAP truncation mutant that lacks the human GPI anchor signal to address two points. Firstly, we wanted to confirm that the endogenous human GPI anchor signal is responsible for the membrane attachment found in recombinant *T. thermophila *strains (see Figure 2, 3 and 4). Secondly, we wanted to investigate if hiAP protein lacking the GPI anchor signal retains activity.

Therefore, we analyzed aliquots of the supernatants after inducing hiAP expression. Significant signals in Western blot analysis were confirmed by phosphatase activity assays. In comparison to the non-induced cells and the wild type control that did not show any alkaline phosphatase activity, we found a significant alkaline phosphatase activity in supernatants of cultures in which the expression of hiAP was induced by the addition of cadmium. As we did not find any endogenous alkaline phosphatase activity in the supernatants the measured intracellular activity in the wild type and non-induced cells is most probably due to a non-secreted, endogenous cytosolic alkaline phosphatase (to compare: see Figure 2B). In conclusion, these data confirm that the GPI anchor signal is responsible for the membrane association of the heterologous expressed full-length hiAP but seems to have little if any influence on its activity. Therefore, hiAP seems to be an ideal reporter enzyme that will allow to analyze protein sorting and secretion in the *T. thermophila *expression system in future.

### Optimization and monitoring of secretion in *T. thermophila*

**Figure 5:**
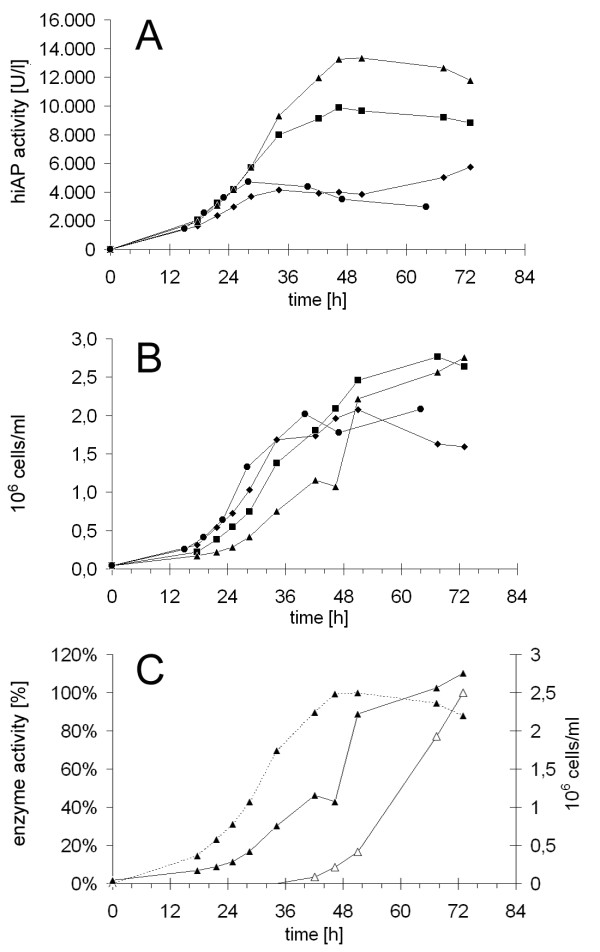
**Analysis of hiAP secretion in *T. thermophila *cultures**. Using the Sixfors bioreactor we analyzed four 500 ml cultures that secrete hiAP without GPI in parallel. The figure shows the activity of extracellular hiAP (A) and the growing curves (B) of four independent fermentations. The cultures were induced with 10 μg/ml (circles, squares, rhombuses) or 15 μg/ml (triangles) cadmium chloride at the beginning of the experiment. Two cultures (triangles, squares) were fed with 10 times concentrated SPP medium at a rate of 2 ml/h. The maximum hiAP activity in the fed cultures (triangles, squares) was reached after 48 h whereas the maximum in the non-fed cultures was reached after 30 h. The fed culture reached about two times more hiAP activity (9,000 U/liter) compared to the unfed culture (4,500 U/liter). The induction with 15 μg/ml cadmium chloride instead of 10 μg/ml together with continuous feeding led to a further 1.5fold increase of the extracellular hiAP activity (14,000 U/liter). In a further fermentation we analyzed the secretion efficiency and compared the hiAP secretion rate to the secretion rate of the endogenous beta-glucosidase (C). The culture grew to a final titer of 2.8 × 10^6 ^cells/ml (solid line, closed triangles). The beta-glucosidase activity started to increase after 36 h until the end of the fermentation (solid line, open triangles) whereas the hiAP activity only increased in the first 48 h of the cultivation reaching a stable level until the end (dashed line, closed triangles), suggesting that the secretion efficiency of the culture and the viability was not influenced by cadmium.

**Figure 6:**
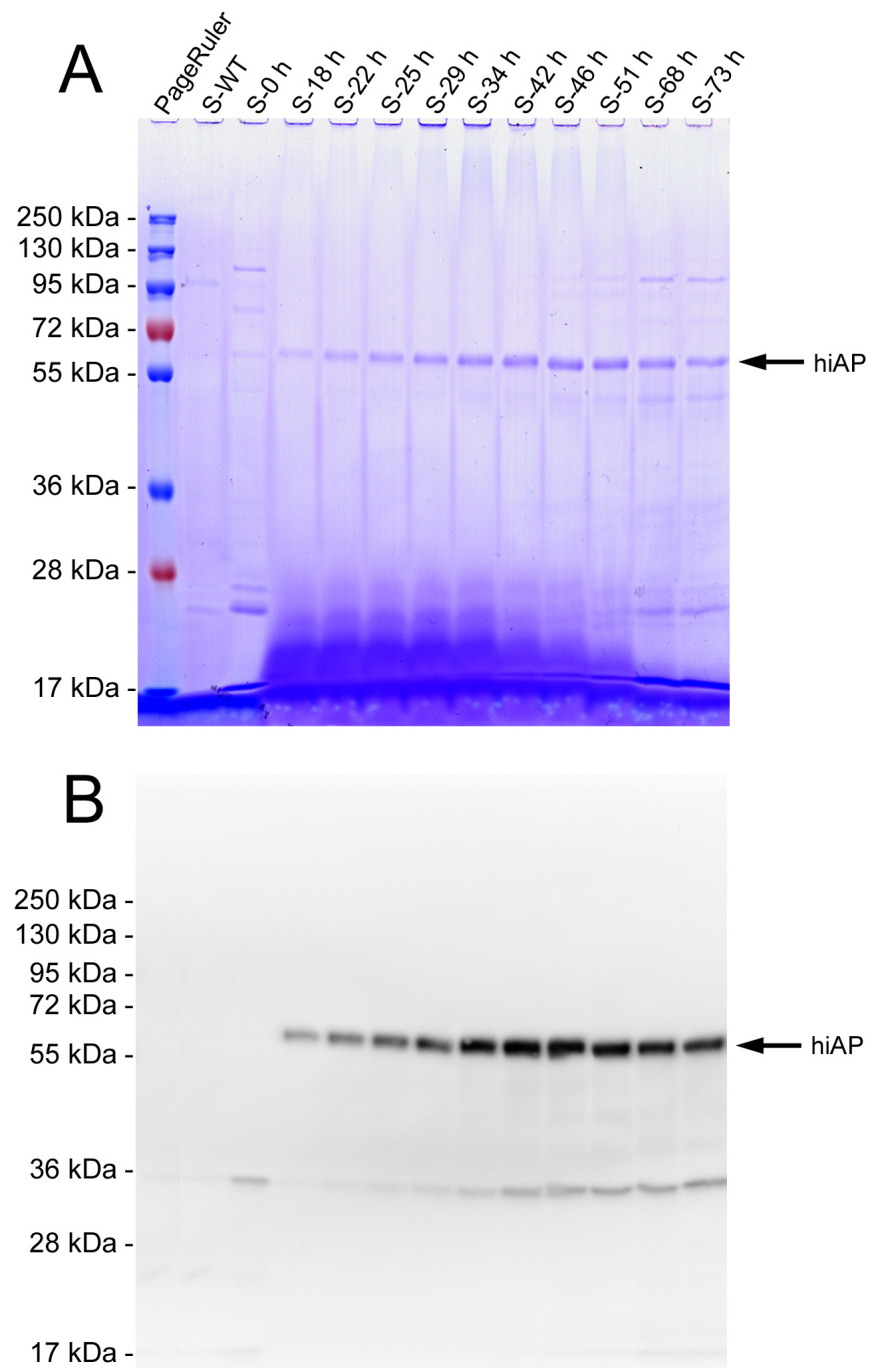
**SDS PAGE of supernatants from different time points during fermentation**. Aliquots of supernatants from different points of time (S-0 h to S-73 h) were analyzed by SDS-PAGE and subsequent Coomassie staining (A) and Western blot (B). In the Coomassie stained gel a prominent band at about 60 kDa became clearly visible which is also detectable by the L-19 antibody (B). No signals were detected before induction (S-0 h) and with a supernatant from a WT culture (S-WT).

Furthermore, the truncated form of hiAP can be used as a technical tool, because it allows to quantify the influence of different fermentation parameters on the yield of expression and secretion. For this purpose, we performed several optimization experiments and tested in how far parameters, like additional medium feeding and multiple cadmium inductions, have an influence on the yield of secreted recombinant hiAP. We performed several independent fermentations using a clone that secretes functional hiAP without the GPI anchor signal (hiAP -GPI). Three independent cultivations were done in parallel to test if multiple inductions with cadmium have an influence on the expression/secretion level. Unexpectedly, we found that multiple cadmium inductions did not lead to a further increase of hiAP activity in the supernatant (data not shown). In contrast to multiple cadmium inductions continuous feeding with concentrated SPP medium during the fermentation process resulted in significantly higher yields of extracellular hiAP (Figure 5A and 6). After 24 h (without feeding) or 48 h (with feeding) the extracellular hiAP activity reached a maximum level and did not increase any further.

We wanted to test if the observed limited time period of hiAP activity increase is due to a reduced secretion activity of the cells. Therefore, we performed a fermentation to measure the beta-glucosidase activity. This enzyme activity usually increases during the late-log and the early-stationary phases [24-28]. We found the highest levels of beta-glucosidase activity in the last 30 hours of the fermentation, indicating that the cells are viable and not inhibited in general secretion.

## Discussion

Here we report the expression of human alkaline phosphatase by the ciliate *T. thermophila*. A codon-adapted artificial gene encoding the full-length precursor enzyme is correctly translated into the functional hiAP enzyme. The heterologous expressed enzyme exhibited phosphatase activity and became N-glycosylated during its passage through the ER/Golgi compartments, suggesting that both, the human signal peptide as well as the human GPI anchor signal, were correctly recognized by the *T. thermophila *protein biosynthesis machinery. Furthermore, the expression cassette encoding the human full-length protein, including the C-terminal GPI anchor showed a membrane associated localization on the surface of the ciliate cells, whereas the C-terminal truncated protein, lacking this GPI anchor recognition signal was secreted into the medium.

The distinct localization pattern of the stained cells (see Figure 3C and Additional file 1 and 2) raises the interesting question which signals determine protein targeting and localization within the ciliates' membranes. Previous results from the Tiedtke group (University of Münster, Germany) showed that hydrolases of the phagolysosomal compartments (e.g. glycosidases, proteinases and lipases) are secreted into the surrounding medium [26,29,30]. *Tetrahymena *possesses three secretory pathways, namely regulated exocytosis of dense-core granules, constitutive secretion of lysosomal enzymes and phagocytosis [31]. At least, the first two of these routings can be used for recombinant protein secretion [10,14]. However, the exact sites of secretion remain to be determined and therefore it is not really known whether or not different secretory and/or surface proteins use different exocytotic pathways.

In previous experiments we could show that fusion proteins of the *T. thermophila *PLA_1 _signal peptide with the human DNase I became secreted and correctly processed [14]. Interestingly, the results we show here clearly indicate that also human signal peptides are sufficient for proper secretion. As recently demonstrated by the Turkewitz group (Department of Molecular Genetics and Cell Biology, University of Chicago) *T. thermophila *provides many excellent features as a model system to analyze regulated secretion in more detail [32,33]. The truncated hiAP used in this study provides an additional instrument to address such questions, because truncated hiAP fused to signal peptides or to parts of proteins that are secreted continuously (*e. g. *lysosomal hydrolases) or in a stimulus dependent manner (*e. g. *proteins of the *GRL *family) might allow deeper insights into the secretory pathways and their regulation. Illustrating the underlying factors that regulate expression and secretion is of paricular importance for high yield recombinant protein expression, because the efficiency of such processes correlates to the yield of biologically active protein. However, scientific studies that focus on basic research rather than on biotechnological aspects will be necessary to elucidate these questions.

We performed fermentation experiments and used the truncated "hiAP without GPI" system to monitor several modifications of the fermentation process. We found that extracellular hiAP activity increased only in the first 24 to 48 h after induction of the *MTT1 *promoter. Of course on the one hand this observation emphasizes that truncated hiAP provides a powerful tool to quantify the secretion efficiency in living ciliate cells by a very sensitive assay. But on the other hand this result raises the question why the hiAP activity reached a stable level although the *T. thermophila *cells grew further to titers of 2.8 × 10^6 ^cells/ml. One reasonable explanation might be that recombinant hiAP is probably increasingly degraded by the ciliates' proteases of the cathepsin L family. This is in agreement with studies that demonstrated that several endogenous hydrolases including proteases accumulate during the late stages in continuous fermentation processes [26,27]. However, the hiAP activity remained stable upon incubation in cell free medium from late log cultures for at least six hours (data not shown). An additional explanation is probably the induction characteristic of the applied *MTT1 *promoter. The promoter of the metallothionein gene *MTT1 *of *T. thermophila *that was first introduced in 2002 can be rapidly activated by simply adding cadmium ions to the growth medium [23,34-37]. Shang *et al. *stated that *MTT1 *mRNA reaches a maximum level at about 45 min after cadmium addition to the culture medium [23]. However, in the same study it was shown that best expression levels of a recombinant antigen which expression was driven by the *MTT1 *promoter were detectable after 9 h of cadmium induction. This observation fits to the finding that highest hiAP expression level lagged behind the published data regarding endogenous *MTT1 *mRNA level and that protein yield decreases after a certain time of induction. Multiple addition of cadmium did not lead to a "pseudo-constitutive" promoter activity in the cultures, suggesting that the inducible effect disappears the longer the fermentation lasts. Furthermore, it has been shown in *T. pyriformis *that a substantial portion of the added cadmium could accumulate inside the cells by chelating metallothioneines [36]. A combination of both effects, protease degradation as well as the adaptation to the cadmium stress is also possible.

Interestingly, the recombinant expression of human alkaline phosphatases shows a further fascinating aspect. Poelstra *et al. *(1997) and Bentala *et al. *(2002) could show that human alkaline phosphatases are able to reduce mortality in mice infected with Gram-negative bacteria [38,39]. Additionally, Heemskerk *et al. *(2009) were able to demonstrate in a phase IIa study that alkaline phosphatase treatment improved renal function in severe sepsis or septic shock patients [40]. These studies show that alkaline phosphatases, usually catalyzing the hydrolysis of monoesters with release of inorganic phosphate, are also capable of reducing toxicity of endotoxins/lipopolysaccharides (LPS), components of the outer membrane of Gram-negative bacteria.

So far only few tools are available to fight against the clinic features of sepsis and the currently available therapies focused on antagonizing the most pro-inflammatory cytokines or by inactivation of the LPS cascade for example by neutralizing anti LPS antibodies [41].

Unfortunately, the studies also revealed that high dosage of alkaline phosphatases are necessary to attenuate LPS toxicity and reduce mortality. As human sources for alkaline phosphatases are limited, different approaches are tried to obtain sufficient amounts of this enzyme to proceed with the clinical tests. Ciliates that secrete large amounts of recombinant human phosphatases might provide a solution for this problem. A general comparison of *Tetrahymena *with common expression systems is given in Additional file 3.

Nam, Ermonval and Sharfstein (2007) demonstrated production levels of 200 U/liter hiAP with CHO cells in a fed-batch bioreactor with a fermentation time of 5 to 6 days [42]. Later, Nam et *al. *(2008) produced 700 - 2,300 U/liter with CHO cells in a fed-batch bioreactor within 5 days [43]. Furthermore, Chen, Chang and Chang (2004) described a 5 liter fermentation of the yeast *Pichia pastoris *with a duration time of at least 5 days and measured 4,000 Units/liter hiAP in the supernatant [44].

We could demonstrate here that up to 14,000 U/liter hiAP could be produced within 2 days fermentation time in a bench top bioreactor. Moreover, it has to be considered that typical steps for a process optimization, like the improvement of the yield by strain optimization and the development of an elaborated fermentation process, has not been undertaken so far. Thus, it should be possible to increase the production of hiAP significantly. Compared to the production methods which have been described in the past, the here used *Tetrahymena *based expression system seems to be an attractive alternative for the production of hiAP.

## Conclusions

The precursor of the human intestinal alkaline phosphatase becomes correctly processed and is expressed as a functional enzyme. These data confirm the high potential of the ciliate based expression system (CIPEX) which offers the chance to develop a biopharmaceutical enzyme used against life-threatening sepsis caused by Gram-negative bacteria. Furthermore, secreted hiAP can be used as an instrument for scientific purposes as well as a reporter enzyme allowing the quantification of measures that are planned to optimize the ciliate expression system.

## Methods

### Constructs

To ensure gene expression in *T. thermophila*, a synthetic hiAP gene was used that was codon optimized according to the *T. thermophila *codon bias that is mainly found in high expressed genes. The synthetic gene that encodes the full-length human intestinal alkaline phosphatase (see Accession Numbers NP_001622 or NM_00163199) including the endogenous signal peptide and the GPI anchor sequence was introduced into the intermediate plasmid (details on intermediate plasmids and primers are available from the authors).

The used expression plasmids are based on the pH4T2 plasmid and were cloned by standard methods. The final expression cassettes carried a 1 kb fragment of the *MTT1 *promoter, the *BTU2 *terminator sequence of the *neo2 *cassette and the artificial hiAP gene encoding the full-length hiAP with (aa 1- 528) or without (aa 1- 503) the GPI anchor signal sequence (see Figure 1) [21-23].

### Strains, cell culture and transformation of *T. thermophila*

Cultivation of the *Tetrahymena thermophila *strains was done in SPP medium [3] and the transformation of conjugating *T. thermophila *cells were performed as previously described [45].

### SDS-PAGE and Western blot analysis

For detection of recombinant hiAP aliquots, transformed cells were lysed in RIPA buffer (150 mM NaCl, 10 mM Tris pH 7.4, 5 mM EDTA, 0.1% SDS, 0.1% Deoxycholate, 1% Triton x-100, proteinase inhibitor complete, Roche). Gained cell extracts and cell-free culture supernatants were resuspended in sample buffer and separated with 10% or 12% SDS-PAGE. The gels were blotted onto nitrocellulose membranes and blocked with PBS containing 0.05% Tween^® ^20 and 5% skimmed milk (PBS-TM). Recombinant hiAP was detected by a polyclonal antibody derived from sheep against an internal region of human PLAP (L-19, Santa Cruz Biotechnology). After washing with PBS/T and applying a monoclonal HRP-conjugated anti goat/sheep antibody, the blots were developed by using chemiluminescence.

### De-glycosylation assay

Samples consisting of 18 μl cell extract or supernatant and 2 μl 10× denaturation buffer containing 5% SDS and 10% 2-mercaptoethanol were boiled 10 min at 100°C. After that 3 μl of 10× G7 de-glycosylation buffer, 3 μl of 10% NP40 and 2 μl of PNGase F (NEB) were added and adjusted to 30 μl (F+ samples). In the corresponding controls no PNGase F was added (F- samples). Aliquots of these reactions were analyzed by SDS-PAGE and Western blot.

### Cell fractionation

For cell fractionation experiments, 1 to 2 ml of *T. thermophila *culture were collected by centrifugation (1,000 × g at room temperature). Cells were resuspended in RIPA buffer without detergent and cracked on ice by sonification. This preparation was centrifuged for 30 min at 4°C and 20,000 × g. The supernatant (S20) was saved. The pellet (P20) was washed twice with RIPA buffer to avoid the presence of cytosolic proteins in the P20 sample and then resuspended in the original volume of the initial sample. Equal aliquots of the cell suspension (total), the pellet (P20) and the supernatant (S20) were analyzed by SDS-PAGE, Western blot and by the corresponding activity assay.

### Immunofluorescence analysis

Cells were fixed with methanol (-20°C) for 30 min and blocked with 0.1% BSA in PBS. Subsequently, the specific polyclonal goat IgG L-19 antibody against hiAP/PLAP was added (dilution 1:200) for 45 min. Cells were washed three times in 0.1% BSA in PBS. The second antibody conjugated with Alexa 546 was added to the cells. After the final washing (three times in 0.1% BSA in PBS) cells were embedded in Mowiol and analyzed by confocal microscopy using a donkey anti goat antibody (dilution 1:200).

### AP activity assay

The hiAP assay was performed in 96 well microtiter plates as follows: 20 μl of sample were mixed with 200 μl of working substrate (32 mM p-Nitrophenyl Phosphate, 8.7 mM MgCl_2_). Kinetic measurement started immediately at 405 nm at 25°C for 5 min with 30 s intervals. Slopes of blanks were subtracted and activity was calculated with a path length of 5 mm and a millimolar extinction coefficient of 18.7 cm^2^/μmole for p-Nitrophenole [41].

### Optimization and fermentation

All optimization experiments were done in a Sixfors bioreactor (Infors AG, Bottmingen) at 500 ml scale. Cells were grown in SPP-based medium with regulation of pH (7.0), pO2 (25%) and temperature (30°C). pO2 was regulated by stirrer speed (500-1,000 rpm) and air flow (0.1-0.5 liter/min).

## Authors' contributions

IA adopted the hiAP assays and performed together with JR all of the fermentation experiments and participated in manuscript drafting. UB cloned the constructs and transformed the ciliates and made most of the Western blots and participated in manuscript drafting. WR participated in the development of the hiAP assays. SL made the immunofluorescence analysis and images. MWWH and LH participated in the conceptual work. TW made the de-glycosylation assays, participated in Western blots, immunofluorescence analysis and the conceptual work and prepared the manuscript. All authors read and approved the final manuscript.

## Supplementary Material

Additional file 1 **Detailed scan of hiAP expressing *T. thermophila *cells**. Example A and B: Here we show two detailed scans through a fixed cell that expresses full-length hiAP. The single images are made in 0.5 μm steps. The first example is the cell that is already shown in Figure 3B and C (white box, detailed image). The arrows clearly demonstrate the surface localization of recombinant hiAP. The second structure is not further characterized, nevertheless it is present in all analyzed cells. Click here for file

Additional file 2 **detailed scan of hiAP expressing *T. thermophila *cells**. Example C and D: Here two further detailed scans are shown that illustrate the surface display of recombinant hiAP in *T. thermophila*. The arrows clearly demonstrate the surface localization of recombinant hiAP. The second (internal) structure is not further characterized, nevertheless it is present in all analyzed cells. Click here for file

Additional file 3 **Comparison of *Tetrahymena thermophila *with established expression systems**. Overview of established expression systems compared to *Tetrahymena thermophila*. from +, insufficiently fulfilled to ++++, completely fulfilled. Click here for file
