# Supplementary figures and images for: Expression, secretion and surface display of a human alkaline phosphatase by the ciliate Tetrahymena thermophila
Source: BMC Biotechnol. 2011 Jan 31;11:11. doi: 10.1186/1472-6750-11-11 (PMC3042934; doi:10.1186/1472-6750-11-11)

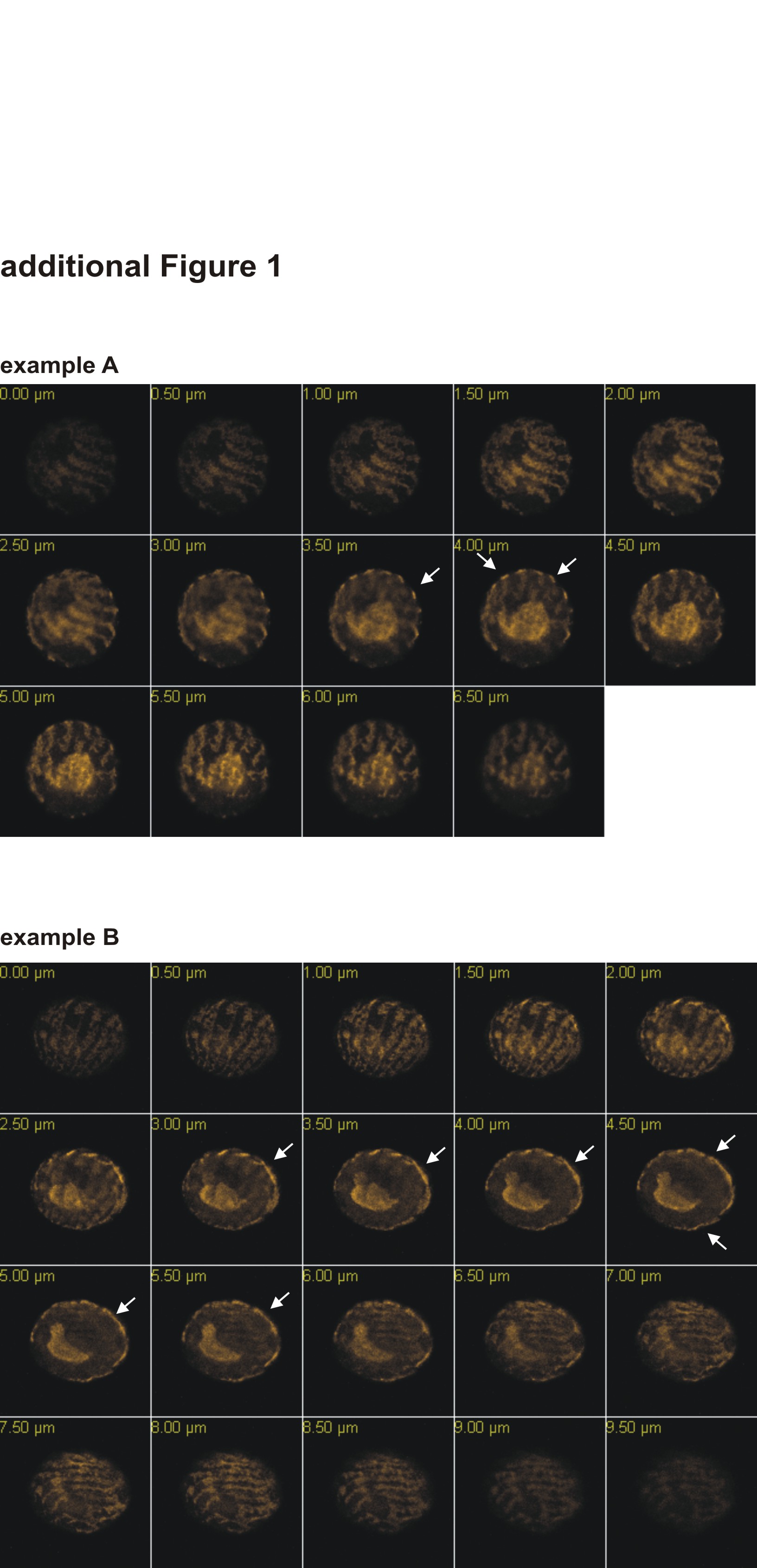

Supplement: Additional file 1 — Detailed scan of hiAP expressing T. thermophila cells. Example A and B: Here we show two detailed scans through a fixed cell that expresses full-length hiAP. The single images are made in 0.5 μm steps. The first example is the cell that is already shown in Figure 3B and C (white box, detailed image). The arrows clearly demonstrate the surface localization of recombinant hiAP. The second structure is not further characterized, nevertheless it is present in all analyzed cells. [file 1472-6750-11-11-S1.JPEG]

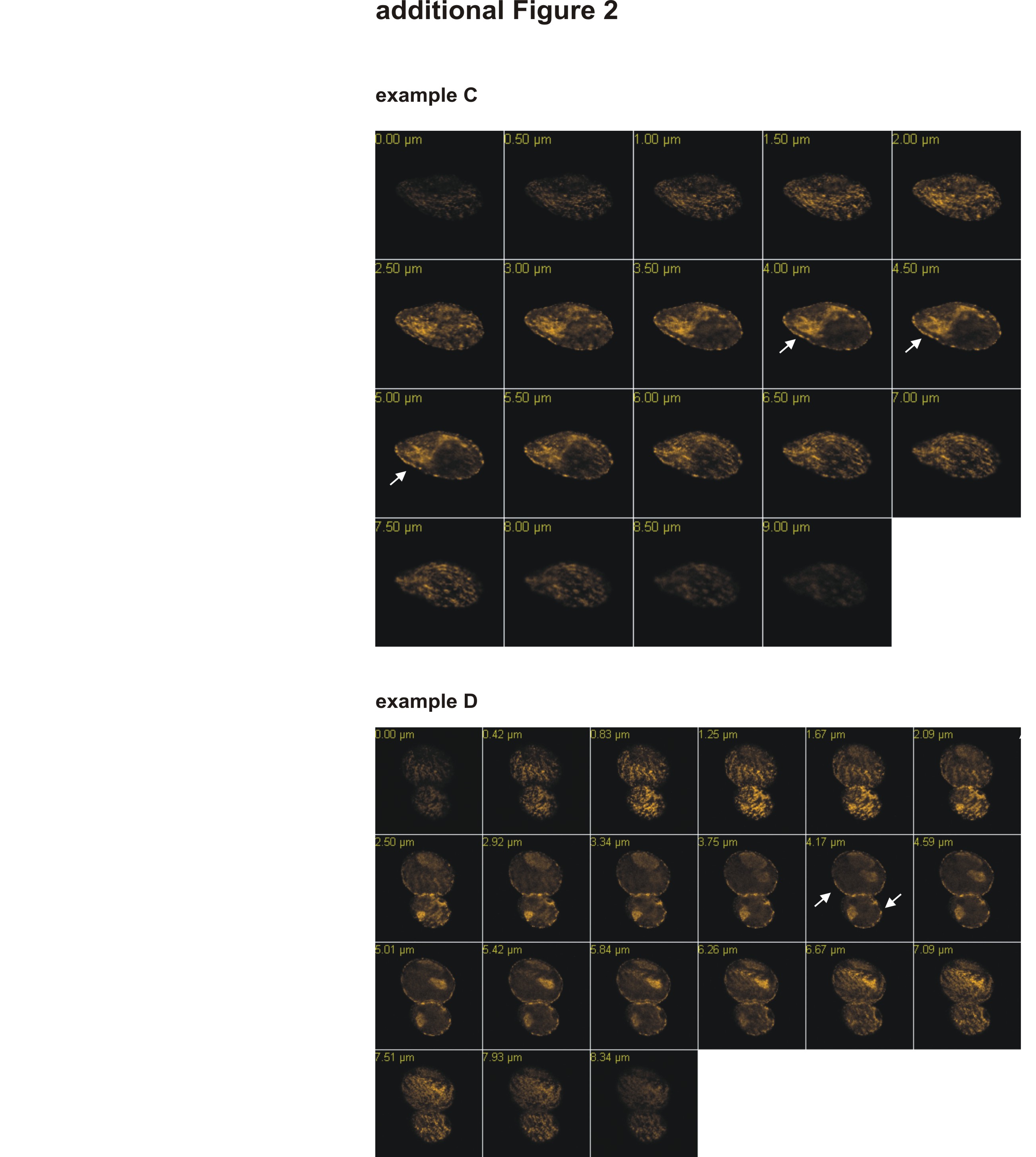

Supplement: Additional file 2 — detailed scan of hiAP expressing T. thermophila cells. Example C and D: Here two further detailed scans are shown that illustrate the surface display of recombinant hiAP in T. thermophila. The arrows clearly demonstrate the surface localization of recombinant hiAP. The second (internal) structure is not further characterized, nevertheless it is present in all analyzed cells. [file 1472-6750-11-11-S2.JPEG]
