# Supplementary material for: Expression, secretion and surface display of a human alkaline phosphatase by the ciliate Tetrahymena thermophila
Source: BMC Biotechnol. 2011 Jan 31;11:11. doi: 10.1186/1472-6750-11-11 (PMC3042934; doi:10.1186/1472-6750-11-11)
Supplement: Additional file 3 — Comparison of Tetrahymena thermophila with established expression systems. Overview of established expression systems compared to Tetrahymena thermophila. from +, insufficiently fulfilled to ++++, completely fulfilled. [file 1472-6750-11-11-S3.DOC]

|  | Transgenic Plants | Transgenic Animals | Mammalian Cells | Insect Cells | Yeast | Bacteria | Ciliates |
| --- | --- | --- | --- | --- | --- | --- | --- |
| Characterization | Novel systems for high volume complex proteins | | Established for complex proteins | Novel system for vaccines | Established for non-complex, glycosylated proteins | Established for non glycosylated proteins | Novel system for complex proteins |
| Glycosylation | Non-human, abnormal, adverse residues | Complex, species and tissue specific, inconsistent | | Abnormal | Adverse glycosylation | Lacking | Oligo-mannose type |
| Reproducibility of Production | ++ | ++ | +++ | ++ | +++ | +++ | Not done |
| Scale-up: Time | ++++ | +++ | + | + | ++ | ++ | ++ |
| Scale-up: Cost | ++++ | +++ | + | + | ++ | ++ | ++ |
| Volume of Production | ++++ | ++++ | ++ | ++ | +++ | +++ | +++ |
| Ethical /political acceptance | - | - | + | + | + | + | + |
| Contamination with human pathogens | No | Yes | Yes | No | No | Yes | No |
| Contamination with Toxins | No | No | No | No | No | Yes | No |
